# The role of the “gut microbiota-mitochondria” crosstalk in the pathogenesis of multiple sclerosis

**DOI:** 10.3389/fmicb.2024.1404995

**Published:** 2024-04-29

**Authors:** Huan Tian, Dunbing Huang, Jiaqi Wang, Huaqiang Li, Jiaxin Gao, Yue Zhong, Libin Xia, Anren Zhang, Zhonghua Lin, Xiaohua Ke

**Affiliations:** ^1^School of Health Preservation and Rehabilitation, Chengdu University of Traditional Chinese Medicine, Chengdu, China; ^2^Department of Rehabilitation Medicine, Shanghai Fourth People's Hospital, School of Medicine, Tongji University, Shanghai, China; ^3^Shengli Clinical Medical College of Fujian Medical University, Fuzhou, China; ^4^Rehabilitation Medicine Center, Fujian Provincial Hospital, Fuzhou, China; ^5^Fujian Provincial Center for Geriatrics, Fujian Provincia Hospital, Fuzhou, China

**Keywords:** multiple sclerosis, gut microbiota, metabolites, mitochondria, prebiotics

## Abstract

Multiple Sclerosis (MS) is a neurologic autoimmune disease whose exact pathophysiologic mechanisms remain to be elucidated. Recent studies have shown that the onset and progression of MS are associated with dysbiosis of the gut microbiota. Similarly, a large body of evidence suggests that mitochondrial dysfunction may also have a significant impact on the development of MS. Endosymbiotic theory has found that human mitochondria are microbial in origin and share similar biological characteristics with the gut microbiota. Therefore, gut microbiota and mitochondrial function crosstalk are relevant in the development of MS. However, the relationship between gut microbiota and mitochondrial function in the development of MS is not fully understood. Therefore, by synthesizing previous relevant literature, this paper focuses on the changes in gut microbiota and metabolite composition in the development of MS and the possible mechanisms of the crosstalk between gut microbiota and mitochondrial function in the progression of MS, to provide new therapeutic approaches for the prevention or reduction of MS based on this crosstalk.

## 1 Introduction

Multiple Sclerosis (MS) is a chronic inflammatory and demyelinating autoimmune disease of the central nervous system (CNS; Filippi et al., 2018), which can result in permanent disability, cognitive impairment, and a significant detrimental impact on a patient’s health-related quality of life (Biernacki et al., 2022). The incidence of MS has continued to increase dramatically in recent years, affecting approximately 3 million patients worldwide (Engelenburg et al., 2022), and it is the most common non-traumatic neurological disorder among young people (McGinley et al., 2021). Multiple sclerosis disease-modifying therapies (DMT) for the treatment of MS have superior efficacy compared to placebo or active comparators, reducing the annual MS relapse rate by 29–68% (McGinley et al., 2021; Charabati et al., 2023), however, DMT has a limited ability to halt disease progression or repair neurological damage. Therefore, there is an urgent need to fully elucidate its pathogenesis and explore new potential therapeutic targets for new MS patients.

As we know, the gut is the largest micro-ecosystem in the human body, containing a total of about 10 to the 14th power microorganisms that support various physiological functions in the body (Wang et al., 2022). Relevant evidence suggests that dysbiosis of gut microbiota is closely related to the pathogenesis of MS (Maglione et al., 2021; Christovich and Luo, 2022) and that gut microbiota can influence the progression of MS through cell-mediated immunomodulatory pathways such as macrophages, Bregs, and Tregs (Hoffman et al., 2023). In addition, the existence of bidirectional gut-brain-axis (MGB) interactions between the gut and the central nervous system plays an important role in MS disease progression (Ghezzi et al., 2021). Although some beneficial or harmful gut microbiota are involved in the pathologic progression of MS, it is still difficult to reveal their contribution to MS pathogenesis.

Mitochondria are highly complex organelles with roles such as synthesizing Adenosine triphosphate (ATP), participating in the metabolism of key compounds, and generating reactive oxygen species (ROS; Blagov et al., 2022; Zhang et al., 2022).In recent years, a great deal of research has been conducted on the possible mechanisms by which mitochondrial dysfunction contributes to the onset or progression of MS (Buonvicino et al., 2023a,b; Wang et al., 2024). Greeck et al. showed that mutations in mitochondrial DNA and reduced activity of the electron transport chain (ETC) lead to degeneration of white and gray matter, exacerbating neurodegeneration and clinical disease progression in all stages of MS (Greeck et al., 2023). In contrast, in others, it was found that enhancing mitochondrial activity in neurons through activation of peroxisome proliferator-activated receptor γ coactivator 1-alpha alpha (PGC-1alpha) prevented neurodegeneration in a mouse model of multiple sclerosis (Rosenkranz et al., 2021). However, the specific mechanisms by which mitochondrial dysfunction affects MS disease are not fully known.

The close interaction between gut microbiota and mitochondria has been comprehensively characterized in a wide range of diseases (Huang et al., 2022; Peng et al., 2022; Zhang et al., 2022; Juárez-Fernández et al., 2023). Metabolite products of gut microbiota play a crucial role in mitochondrial reactive oxygen species, kinetics, and autophagy. However, the specific mechanisms by which crosstalk between gut microbiota and mitochondria influences MS progression are unknown. Therefore, this paper aims to explore the possible mechanisms of gut microbiota and mitochondrial crosstalk in MS progression by analyzing the existing literature and suggesting potential therapeutic strategies that may treat MS.

## 2 A link between MS and gut microbiota

### 2.1 Alteration of gut microbiome in MS individuals

Many studies have discussed the altered gut microbiota regarding abundance and structural composition, and sequencing-based analyses have revealed a different composition of the gut microbiota in MS patients (Table 1).

**Table 1:** Characteristics of intestinal flora in MS patients.

Study ID	Participants	Intestinal flora diversity	Upregulated microbiota	Downregulated microbiota	Sequencing method
Cantarel et al. (2015)	Patient:15 Healthy Patient:8	–	*Bacteroidaceae*, *Faecalibacterium*	*Ruminococcus*	16S rRNA
Miyake et al. (2015)	Patient:20 Healthy Patient:40	α diversity index was not statistically significant	*Anaerostipes* sp., *Faecalibacterium*, *Bacteroides*, *Prevotella*	*Bifidobacterium*, *Streptococcus*	16S rRNA (V1-V2 region)
Chen et al. (2016)	Patient:31 Healthy Patient:36	α diversity index was not statistically significant	*Adlercreutzia*, *Prevotella*, *Parabacteroides*	*Psuedomonas*, *Mycoplana*, *Haemophilus*, *Blautia*, *Dorea*	16S rRNA (V3-V5 region)
Jangi et al. (2016)	Patient:60 Healthy Patient:43	α diversity index was not statistically significant	*Butyricimonas*	*Akkermansia*, *Methanobrevibacter*	16S rRNA (V3-V5 region)
Tremlett et al. (2016)	Patient:18 Healthy Patient:17	α diversity index was not statistically significant	*Lachnospiraceae*, *Ruminococcaceae*	*Bifidobacterium*, *Desulfovibrionales*, *Christensenellaceae*	16S rRNA (V4 region)
Cekanaviciute et al. (2017)	Patient:71 Healthy Patient:71	α diversity index was not statistically significant	*Parabacteroides*	*Acinetobacter*, *Akkermansia*	16S rRNA (V3-V5 region)
Cosorich et al. (2017)	Patient:19 Healthy Patient:17	α diversity index was not statistically significant	*Prevotella*	-	-
Forbes et al. (2018)	Patient:19 Healthy Patient:23	α diversity index was statistically significant	*Lachnospiraceae*, *Gemmiger* sp.*, Sporobacter*	*Actinomyces*, *Clostridium III*, *Eggerthella*, *Faecalicoccus*, *Streptococcus*	16S rRNA (V4 region)
Kozhieva et al. (2019)	Patient:15 Healthy Patient:15	α diversity index was statistically significant	-	*Actinomycetales*, *Desulfovibrionales*, *Ruminococcaceae*, *Verrucomicrobiales*, *Gemmiger sp*	16S rRNA (V3-V4 region)
Oezguen et al. (2019)	Patient:13 Healthy Patient:14	α diversity index was not statistically significant	*Succinivibrio*	*Butyricicoccus*, *Clostridium III*, *Coprococcus*, *Escherichia*/*Shigella*, *Ruminococcus*, *Dorea*, *Parabacteroides*, *unclassified Coriobacteriaceae*	16S rRNA (V3-V5 region)
Storm-Larsen et al. (2019)	Patient:36 Healthy Patient:165	α diversity index was not statistically significant	*Faecalibacterium*	-	16S rRNA (V3-V4 region)
Ventura et al. (2019)	Patient:45 Healthy Patient:44	α Diversity index was not statistically significant	-	*Akkermansia*	16S rRNA (V4 region)
Zeng et al. (2019)	Patient:34 Healthy Patient:34	α diversity index was not statistically significant	*Prevotella*	*Streptococcus*	16S rRNA (V3-V4 region)
Ling et al. (2020)	Patient:22 Healthy Patient:33	α diversity index was not statistically significant	*Bilophila*, *Butyricicoccus*, *Clostridium III*, *Faecalibacterium*, *Haemophilus*, *Dorea*, *Roseburia*, *Gemella*, *Granulicatella*	*Flavonifractor*, *Blautia*	16S rRNA (V3-V4 region)
Choileáin et al. (2020)	Patient:26 Healthy Patient:39	The Shannon index was significantly reduced in MS patients	*Clostridium III*, *Coprococcus*, *Paraprevotella*, *Methanobrevibacter*	*Ruminococcaceae*	16S rRNA (V4 region)
Reynders et al. (2020)	Patient:98 Healthy Patient:120	α diversity index decreased	*Butyricicoccus*	*Akkermansia*, *Clostridium III*, *Parabacteroides*, *Gemmiger* sp.*, Sporobacter*	16S rRNA (V4 region)
Saresella et al. (2020)	Patient:38 Healthy Patient:38	α diversity index was not statistically significant	*Coprococcus*, *Blautia*, *Parabacteroides*, *Roseburia*	*Collinsella*, *Eubacterium*	16S rRNA (V3-V4 region)
Castillo-Álvarez et al. (2021)	Patient:15 Healthy Patient:14	α diversity index was not statistically significant	*Eubacterium*, *Prevotella*, *Uncultured Bacteroides*, *Uncultured alpha Proteobacterium*, *Uncultured Pseudomonas sp*	*Alistipes onderdonkii*, *Anaerostipes* sp. *Clostridium III*, *Coriobacterium* sp., *Faecalibacterium*, *Ruminococcus*, *Bifidobacterium*, *Uncultured Oscillospiraceae bacterium*, *Uncultured Blautia* sp.*, Uncultured Sinorhizobium* sp., *Uncultured Dialister sp*	16S rRNA (V4 region)
Galluzzo et al. (2021)	Patient:15 Healthy Patient:15	α diversity index was not statistically significant	*Bacteroidaceae*, *Lachnospiraceae*, *Tannerellaceae*, *Rikenellaceae*	*Akkermansiaceae*, *Clostridiales*, *Desulfovibrionaceae*, *Family XIII*, *Christensenellaceae*, *Ruminococcaceae*	16S rRNA (V3-V4 region)
Takewaki et al. (2020)	Patient:118 Healthy Patient:55	α diversity index was not statistically significant	*Alistipes onderdonkii*, *Megamonas*, *Roseburia*	*Bifidobacterium*, *Streptococcus*	16S rRNA (V1-V2 region)
Cox et al. (2021)	Patient:243 Healthy Patient:40	α diversity index was not statistically significant	*Anaerococcus*, *Blautia*, *Dorea*	*Akkermansia*, *Clostridaceae*, *Clostridium III*, *Enterobacteriaceae*, *Ruminococcaceae*	16S rRNA (V4 region)
Cantoni et al. (2022)	Patient:24 Healthy Patient:25	α diversity index was not statistically significant	*Anaerostipes* sp., *Faecalibacterium*, *Prevotella*, *Lachnospiraceae*	-	16S rRNA (V1-V3 region), Metagenomic whole-genome shotgun approach
Mekky et al. (2022)	Patient:30 Healthy Patient:20	-	*Prevotella*	***B. fragilis***, *C. perfringes*	16S rRNA
Vacaras et al. (2023)	Patient:50 Healthy Patient:21	α diversity index was not statistically significant	*Faecalibacterium*, *Prevotella*, *Bifidobacterium*	-	16S rRNA (V3-V4 region)

This paper summarizes 24 studies on MS involving a total of 1,075 MS patients and 947 healthy controls (HC; Cantarel et al., 2015; Miyake et al., 2015; Chen et al., 2016; Jangi et al., 2016; Tremlett et al., 2016; Cekanaviciute et al., 2017; Cosorich et al., 2017; Forbes et al., 2018; Kozhieva et al., 2019; Oezguen et al., 2019; Storm-Larsen et al., 2019; Ventura et al., 2019; Zeng et al., 2019; Choileáin et al., 2020; Ling et al., 2020; Reynders et al., 2020; Saresella et al., 2020; Takewaki et al., 2020; Castillo-Álvarez et al., 2021; Cox et al., 2021; Galluzzo et al., 2021; Cantoni et al., 2022; Mekky et al., 2022; Vacaras et al., 2023). Although the differences in gut microbiota properties between the MS and HC groups were not found to be statistically significant in animal and human studies on MS. However, the relative abundance of *Akkermansia*, *Christensenellaceae*, *Desulfovibrionales*, *Ruminococcus*, and *Streptococcus* in the MS group was significantly higher than in (HC) group. One study found that *Akkermansia* isolated from MS patients reduced RORγt^+^ and IL-17 γδT cells to improve experimental autoimmune encephalomyelitis (EAE; Cox et al., 2021) Conversely, *Akkermansia muciniphila* may contribute to exacerbation of chronic inflammation and exacerbation of MS symptoms either directly by shifting the immune response to the Th1 phenotype or indirectly by interacting with other bacteria and reducing the ability to differentiate against Treg (Cekanaviciute et al., 2018). *Christensenellaceae* is influenced by the genetic composition of the host in the gut microbiome (Goodrich et al., 2014) and is a potential indicator of the risk of mortality in neurocritical care patients (Xu et al., 2019). *Ruminococcus* is a genus that produces abundant propionic and butyric acids and can participate in the digestion of food and the reduction of inflammatory factors with the maintenance of intestinal barrier function (Crost et al., 2023). Both *Clostridium butyricum* and norfloxacin treatment reduced the abundance of *Desulfovibrionales* and *Ruminococcus*, which inhibited the Th17 cell response promoted Treg differentiation, and attenuated the severity of EAE in mice through mitogen-activated kinase (MAPK) signaling (Chen et al., 2019). *Streptococcus* abundance has been shown to positively correlate with the proportion of Th17 cells and negatively correlate with Tregs and is therefore hypothesized to be a potential key factor in the development and/or relapse of MS disease (Zeng et al., 2019).

In contrast, the relative abundance of *Bacteroidaceae*, *Lachnospiraceae*, *Prevotella,* and *Roseburia* was significantly lower in the MS group than in the HC group. Studies on the diet of EAE mice found that intermittent fasting (IF) increased intestinal *Bacteroidaceae* abundance, decreased IL-17 cell production, increased Tregs differentiation, and ameliorated the clinical course and pathology of EAE (Cignarella et al., 2018). *Lachnospiraceae* were shown to impair oligodendrocyte differentiation in cultured cells and were associated with impaired myelin formation in mice (Katz Sand et al., 2019). Iljazovic et al. found that *Prevotella* colonization led to changes in the metabolism of the gut microbiota, which reduced the production of IL-18, thereby exacerbating intestinal inflammation and leading to systemic autoimmunity (Iljazovic et al., 2021). In a mouse model, *Prevotella histicola* reduces pro-inflammatory Th1 and Th17 cells in the CNS by inducing the number of CD4^+^FoxP3^+^ regulatory T-cells thereby reducing the severity of EAE (Shahi et al., 2019). *Roseburia* spp. plays an important role in the regulation of barrier homeostasis, immune cells, and cytokine release through its metabolite, butyrate. Thus, *Roseburia* also plays an important role in alleviating inflammation in autoimmune diseases (Nie et al., 2021).

The controversy over the abundance of *Blautia*, *Dorea*, *Faecalibacterium, Methanobrevibacter, Parabacteroides*, and *Ruminococcaceae* in MS and HC groups is consistent with the findings of a systematic review and review, probably due to significant differences in disease severity and duration, as well as differences in changes in gut microbiota (Ordoñez-Rodriguez et al., 2023).

Based on these results, it is suggested that interventions or changes in gut microbiota can alter susceptibility to inflammatory demyelination in MS in humans or animals and that gut microbiota can be an important hub for regulating and synchronizing many of the changes associated with the pathophysiology of MS.

### 2.2 Potential mechanisms of gut microbiota metabolites in MS

Bidirectional interactions involving endocrine, neuroimmune, and chemical interactions between the gut microbiota and the central nervous system form the MGB (Ullah et al., 2023). Gut microbiota metabolites are involved in the maintenance of homeostasis in the body and influence the development of MS through the MGB (Table 2), and the following description highlights the most important metabolites of the gut microbiota that have recently been involved in the progression of MS.

**Table 2:** A summary of current studies about the impact of gut microbial metabolites in MS *in vivo* and *in vitro*.

Metabolites	Human/animal/cell type species	Conclusions	Reference and year
bile	MS patients	a decrease in the abundance of microbes involved in fatty acid metabolism (bile metabolism)	Chen et al. (2016)
Trp	MS patients	The QA/KA ratio was higher in MS groups compared to controls	Lim et al. (2017)
Trp	MS patients	each 1 mcg/mL increase in serum Trp level was associated with a 20 and 32% decrease in adjusted odds of having MS.	Nourbakhsh et al. (2018)
SCFAs (acetate, propionate, and butyrate)	MS patients	A depletion of acetate, propionate, and butyrate was observed in MS. SCFA level was positively correlated with pTreg frequency.	Zeng et al. (2019)
SCFAs (butyrate)	C57BL/6 J (B6) mice	butyrate treatment suppressed demyelination and enhanced remyelination	Chen et al. (2019)
butyricum	C57BL/6 J WT mice	*C. butyricum* treatment reduced Th17 response and increased Treg response in the gastrointestinal tract and extra-gastrointestinal organ systems in EAE mice.	Chen et al. (2019)
SCFAs (acetate, butyrate, and valerate)	MS patients	In MS patients, butyrate was higher and acetate lower. Acetate levels are associated negatively with IFNG. IFNG and TNF were favorably linked with butyrate and valerate.	Olsson et al. (2021)
SCFAs (acetate, propionate, and butyrate)	MS patients, IL-10−/− mice	SCFAs (acetate, propionate, and butyrate) are significantly decreased in MS. SCFAs increase IL-10 T cells, Th17 and Th1 effector cells	Park et al. (2019)
SCFAs (propionate)	MS patients	MS exhibited reduced PA amounts. T cells increased, whereas Th1 and Th17 cells decreased after PA intake.	Duscha et al. (2020)
SCFAs (acetate)	MS patients	acetate levels were higher in patients, and an inverse correlation exists between acetate levels and naïve CD4+ T lymphocytes, while a direct correlation exists with IL-17-producing CD8+ T cells.	Pérez-Pérez et al. (2020)
SCFA (butyric acid (BA), Caproic acid (CA))	MS patients	In MS, the concentration of BA was reduced and that of CA was increased. The higher plasma concentrations of lipopolysaccharide and intestinal fatty acid-binding protein. CA was positively associated with CD4+/IFNγ+ T lymphocytes, and the BA/CA ratio correlated positively with CD4+/CD25high/Foxp3+ and negatively with CD4+/IFNγ+ T lymphocytes.	Saresella et al. (2020)
SCFA (butyrate)	MS patients	The microorganisms that produce butyric acid are reduced in MS. The abundance of butyrate correlated positively with the levels of chemokines such as IL-8 and MIP-1a, while it correlated negatively with those of inflammatory cytokines such as TNF-α.	Ling et al. (2020)
Trp	MS patients	MS patients had a significantly lower urine concentration of kynurenine than healthy controls.	Gaetani et al. (2020)
Bile acids (TUDCA)	MS patients	Multiple bile acid metabolites were found in smaller amounts in MS, and TUDCA supplementation ameliorated neuroinflammation in EAE through its effects on GPBAR1.	Bhargava et al. (2020)
SCFAs (propionate acid)	MS patients	An increase of functionally competent regulatory T (Treg) cells in MS, whereas Th1 and Th17 cells decreased significantly after PA intake.	Duscha et al. (2020)
SCFAs (butyrate and propionate)	MS patients	A reduction in butyrate and propionate biosynthesis and corresponding metabolic changes in MS.	Takewaki et al. (2020)
AAA metabolites	MS patients	AAA metabolism results in the reduced production of immunomodulatory metabolites and increased production of metabotoxins in MS.	Fitzgerald et al. (2021)
SCFAs (butyrate, valerate)	MS patients	MS patients had increased butyrate levels, and butyrate and valerate correlated positively with proinflammatory cytokines (IFNG and TNF). SCFAs were inversely connected with clinical impairment.	Olsson et al. (2021)
SCFAs (butyrate)	MS patients	MS has reduced indolelactate and its producing bacteria and lower levels of species and genes involved in butyrate production.	Levi et al. (2021)
SCFAs (butyrate)	C57BL/6JOlaHsd mice	the butyrate was increased and ameliorated the Clinical Signs and Reduced CNS Autoimmunity in EAE Mice	Calvo-Barreiro et al. (2023)
SCFAs (propionate)	MS patients	the propionate was reduced in MS patients	Trend et al. (2021)
LPS	MS patients	MS participants had higher breakdown of LPS molecules, but lower resistant starch metabolism.	Mirza et al. (2022)
SCFAs	MS patients	MS patients had decreased SCFAs levels	Cantoni et al. (2022)
tryptophan metabolism	PWD/PhJ (PWD) mice	The number of IL-17 and IFNγ-producing CD4+ T cells was also reduced with low tryptophan, *L. reuteri* colonization elevated the number and frequency of TCRγδ cells and their production of IL-17, in the presence of high dietary tryptophan.	Montgomery et al. (2022)
Bile acids (TUDCA)	C57BL/6 mice	TUDCA suppressed excessive activation of astrocytes and inhibited inflammatory responses in the cerebral cortex of EAE mice via TGR5/AKT/NFκB signaling pathway.	Xu et al. (2023)
Bile acids (TUDCA)	MS patients	Central memory CD4+ and Th1/17 cells decreased, while CD4+ naïve cells increased in the TUDCA arm compared to placebo.	Ladakis et al. (2024)

Trp, altered tryptophan; SCFAs, short-chain fatty acids; AAA, aromatic amino acid; LSP, lipopolysaccharide; TUDCA, effects of a secondary bile acid; GPBAR1, G protein-coupled bile acid receptor.

EAE and cuprizone (CPZ) is a commonly used animal model for MS (Ji et al., 2024). Regulatory T cells (Tregs) interacting with B cells and CNS glial cells are key factors in MS and EAE neurodegeneration (Brummer et al., 2022). Among them, helper T cells (Th)1 and Th17 cells are the main coordinators of pathogenic inflammatory processes in MS (Kamali et al., 2019). During pro-inflammatory cytokine production, self-antigen presentation to T cells acts as a pathogenic B-cell effector in MS. In EAE, antigen presentation by B cells is sufficient to cause disease by predominantly reactivating Th1 and Th17 cells (Thomann et al., 2023).

Altered gut microbiota in MS patients can be attributed to T-cell dysfunction (Hussman et al., 2016). Recent studies have shown that gut microbiota metabolites such as short-chain fatty acids (SCFAs), secondary bile acids, and tryptophan (Trp) can act as immunomodulators of regulatory T cells and T helper cells (Calvo-Barreiro et al., 2023), Important regulators of brain and neuronal activity that can influence MS progression (Tan et al., 2023).

SCFAs composed of acetic acid, propionic acid, and butyric acid act as a link between the gut microbiota and the immune system and can protect against many autoimmune diseases by regulating T cell differentiation (Liu et al., 2024). Reinforcement of the blood–brain barrier (BBB) by SCFAs suppresses experimental CNS inflammation (Kim, 2023). Previous studies have shown lower levels of SCFAs in the feces of MS patients (Cantoni et al., 2022), SCFAs positively correlate with Treg levels (Zeng et al., 2019). SCFAs increase the number of regulatory T cells (Park et al., 2019), and decrease clinical activity in MS patients (Park et al., 2019). Lower levels of acetic acid in feces and plasma were observed in MS patients in Park et al. (2019) and Zeng et al. (2019). In a study by Pérez et al. acetate levels were found to be negatively correlated with CD4+ Treg cells and positively correlated with CD8+ Treg cells (Pérez-Pérez et al., 2020). Not only that, MS patients had lower levels of propionic acid in feces and plasma (Park et al., 2019; Zeng et al., 2019; Duscha et al., 2020; Takewaki et al., 2020; Trend et al., 2021), but MS patients continued to have a significant increase in T-cell counts and a decrease in Th1 and Th17 cell counts after 2 weeks of propionic acid ingestion. Propionic acid ingestion for 3 years reduced MS annual relapses and stabilized the disease (Duscha et al., 2020). Similarly, fecal and plasma levels of butyric acid have been found to be reduced in MS patients in several studies (Park et al., 2019; Zeng et al., 2019; Ling et al., 2020; Saresella et al., 2020; Takewaki et al., 2020), and *Clostridium butyricum* treatment decreased Th17 responses and increased Treg responses in EAE mice (Chen et al., 2019). And elevated fecal and plasma caproic acid (Saresella et al., 2020). Caproic acid was positively correlated with CD4+/ nterferon-γ (IFNγ) + T cells, and the butyric acid/caproic acid ratio was positively correlated with CD4+/CD25high/Foxp3+ and negatively correlated with CD4+/IFNγ+ T lymphocytes (Saresella et al., 2020). However, there is disagreement about acetic and butyric acid levels in MS, and Pérez et al. examined SCFA levels in plasma from Spanish patients and found a significant increase in acetic acid levels (Pérez-Pérez et al., 2020). Olsson et al. studied 58 patients and found a significant elevation of butyric acid in their plasma (Olsson et al., 2021).

The results of recent animal studies have shown that the gut metabolite bile acids, taurine deoxycholic acid (TUDCA), has a good therapeutic advantage in neurological disorders due to its neuroprotective properties, nontoxic nature, and ability to cross the blood–brain barrier (Grant and DeMorrow, 2020). Bhargava et al. performed cellular experiments and found that bile acid receptor expression was enhanced in immune and neuroglial cells from MS patients, and supplementation of cells and mice with TUDCA revealed that TUDCA alleviated symptoms in MS patients by modulating the bile acid receptor G protein-coupled bile acid receptor 1 (GPBAR1; Bhargava et al., 2020). TUDCA was found to inhibit inflammatory responses in C57BL/6 mice by suppressing astrocyte hyperactivation in the cerebral cortex of EAE mice through the G-protein coupled bile acid receptor Gpbar1/ threonine kinase/ I (TGR5/AKT/NF-KB) signaling pathway (Xu et al., 2023). Another study also found that TUDCA supplementation increased circulating levels of several bile acids, affected peripheral immune phenotypes, and altered gut microbiota (Ladakis et al., 2024). In a mouse model of EAE, agonism of GPBAR1 was found to reduce the number of monocytes and T cells in the CNS and to reduce monocyte and microglia activation in the CNS as well as monocyte activation in the periphery, and a reduction in the severity of the disease in EAE was found to be detected by clinical scores (Lewis et al., 2014).

In addition to SCFA and bile acids, elevated gut metabolite Trp is associated with multiple sclerosis compared to healthy controls (Nourbakhsh et al., 2018). EAE animal studies found that the number of IL-17- and IFNγ-producing CD4+ T cells was reduced at low tryptophan levels, whereas colonization of *Lactobacillus reueri*at high tryptophan levels increased the number and frequency of TCRγδ cells and increased IL-17 production, suggesting that *Lactobacillus reueri*tryptophan metabolism promotes host susceptibility to CNS autoimmunity (Montgomery et al., 2022). Metabolism of the canine uridine pathway is an important pathway for Trp involvement in the regulation of immunity, neurological function, and intestinal homeostasis (Platten et al., 2019), Includes kynurenine (KA) and quinolinic acid (QA) as neuroactive kynurenine pathway metabolites with neuroprotective and neurotoxic properties respectively (Zheng et al., 2022). A study in Italy found that urinary kynurenine concentrations in *MS* patients were significantly lower than in the HC group (Gaetani et al., 2020). T-cell suppression mediated by IDO-1 in MS patients leads to MS progression through the production of excitotoxic QA (and increased QA/KA ratio) by infiltrating macrophages (Lim et al., 2017).

Other gut metabolites such as changes in the metabolism of aromatic amino acids (AAA) in MS patients, the AAA-derived lactate metabolite ILA reduces pro-inflammatory cytokine interleukin-6(IL-6) and IL-1β production and ameliorates worsening MS severity (Fitzgerald et al., 2021). Lipopolysaccharide (LPS) catabolism was higher in MS participants compared to HC group (Mirza et al., 2022). In EAE mice, LPS-induced dendritic cell (DC) apoptosis inhibits Th17 and IFN-γ cell activity and blocks myelin oligodendrocyte glycoprotein (MOG)-triggered CD4+ T-cell activation *in vivo*, thereby inhibiting the development of EAE (Zhou et al., 2014).

In summary, there is growing evidence that gut microbiota and their metabolites can communicate with the host via T cells and that T cell-mediated immune pathways play a large role in controlling the host immune system and inflammation. We believe that changes in metabolites from gut microbiota may be a key way to understand how gut microbiota and MS “talk to each other.” However, there is still much uncertainty about this issue.

#### 2.2.1 Effect of mitochondria on MS

The occurrence of mitochondrial abnormalities has been demonstrated in studies of MS patients as well as in various animal models of MS. These abnormalities include accumulation of reactive ROS, altered mitochondrial dynamics, and mitochondrial autophagy (Alshial et al., 2023).

Specific neuronal cells in the CNS of MS patients (e.g., astrocytes, activated macrophages, and microglia) produce reactive ROS, and mitochondrial dysfunction increases the level of ROS, which leads to neuronal and glial cell damage (Friese et al., 2014). Peripheral Blood Nucleated Cells Obtained from MS Patients Show Lower Reductive Activity and Increased ROS Production (Pistono et al., 2020). Yang et al. found that PGC-1α interacted with transcription factor silencing information regulator 2 homolog 1 to maintain the deacetylated state of PGC-1α, which not only inhibited the production of reactive ROS but also inhibited the activation of microglia and astrocytes, resulting in anti-inflammatory effects in EAEs (Yang et al., 2024). Alissafi et al. found that inhibition of ROS in regulatory T cells of EAE mice by MitoTEMPO attenuates the DNA damage response and avoids Treg cell death, thereby inhibiting the development of Th1 and Th17 cells (Alissafi et al., 2020), Inhibition of Th1 and Th17 cells which is protective in against EAE (Li Y. et al., 2022; Angelini et al., 2023).

Mitochondrial dynamics for mitochondrial fission and fusion can regulate changes in number, morphology, and localization within cells (Tilokani et al., 2018). Disturbances in mitochondrial dynamics may affect many cellular and molecular pathways such as calcium-dependent immune activation, transcription factor phosphorylation cytokine secretion, and even cell death (de Oliveira et al., 2021). Ion channel blockers found in animal models of EAE block voltage-gated Ca^2+^ channels can alleviate MS symptoms (Ulshöfer et al., 2022). DE et al. found that increased mitochondrial DNA damage in MS, which in turn caused upregulation of mitochondrial reverse transcriptase activity, led to excessive release of inflammatory mediators and neuronal damage. In addition, progesterone treatment improved mitochondrial ultrastructure and increased fission/fusion protein mRNA, enhancing neuroprotective and anti-inflammatory effects in EAE mice (De Nicola et al., 2018). Activation of Drp1 (a mitochondrial fission protein) in oligodendrocytes by tumor necrosis factor-α (TNFα) or ROS treatment in oligodendrocytes in EAE versus CPZ animal models, and inhibition of Drp1 activation by P110 (an inhibitor that prevents Drp1 from binding to mitochondrial fission proteins) attenuates mitochondrial fragmentation and cell death in oligodendrocytes, alleviates disease severity in EAE mice, oligodendrocyte loss and myelin sheath injury can also reduce oligodendrocyte loss and myelin damage in CPZ models (Lei and Lin, 2024).

Autophagy is a conserved intracellular degradation pathway, and the conclusion that mitochondrial dysregulation is the determinant of MS autophagy in animal models of MS has also been demonstrated *in vivo* (Li et al., 2019). The study found the presence of excess mitophagy markers in the cerebrospinal fluid (CSF) and serum of patients with MS (Patergnani et al., 2018). Direct activation of the mitochondrial autophagy mechanism has been demonstrated in an *in vivo* demyelinating mouse model. Studies have shown that antipsychotic compounds haloperidol and clozapine (identified as potential autophagy inhibitors) block aberrant mitophagy and reduce TNF-α and IL-1-β production, prevent demyelination in CPZ models, induce remyelination, and restore MS behavioral deficits (Patergnani et al., 2021).

It has been found that enhancing mitochondrial autophagic clearance and inducing autophagosome formation can play a protective role in MS animal models (Yang et al., 2024). Moreover, the deletion of autophagy-related gene 7 (Atg7) in DCs can reduce the *in vivo* activation of T cells, which in turn improves EAE (Bhattacharya et al., 2014).

### 2.3 Gut microbiota and mitochondrial interactions

Recent evidence from microbiology suggests that mitochondria originate with the phylum α-Ascomycetes and that there is a close connection between gut microbiota and mitochondria, despite the different roles of mitochondria and gut microbiota (Bajpai et al., 2018; Murphy and O’Neill, 2024). Bidirectional communication exists between gut microbiota and mitochondria, and gut microbiota has become a dynamic central regulator of mitochondrial function whereas mitochondria are the most responsive organelles to microbial signaling (Jackson and Theiss, 2020).

Gut microbiota and their metabolites can regulate mitochondrial energy metabolism, ROS production, and immune-inflammatory responses by participating in the Borbolis et al. (2023) and Endres and Friedland (2023). Li et al. found that butyrate can induce mitochondrial autophagy through the activation of adenylate-activated protein kinase (AMPK), thereby attenuating hydrogen peroxide- (H_2_O_2_-)induced oxidative stress, intestinal epithelial barrier damage, and mitochondrial dysfunction (Li X. et al., 2022). Alterations in butyrate may affect glial cells, leading to mitochondrial dysfunction in microglia and oligodendrocytes, which up-regulates oxidants and reduces their ability to cope with oxidants, thereby inhibiting myelin synthesis in MS disease (Anderson et al., 2019). MS patients ingesting propionic acid for 2 weeks showed a significant increase in Treg cells and a significant decrease in Th1 and Th17 cells, restoring mitochondrial respiration in MS (Duscha et al., 2020). Therefore, it is hypothesized that propionic acid may ameliorate MS symptoms by modulating mitochondrial function in T cells. In studies of atherosclerotic disease, it has been found that short-chain fatty acids, trimethylamine, and bile acids can affect mitochondrial DNA (mtDNA) mutations, oxidative stress, and alterations in mitochondrial autophagy, thereby increasing inflammation, further disrupting intracellular homeostasis and innate immune homeostasis, and exacerbating atherosclerosis (Li et al., 2023). Dysbiosis of gut microbiota causes translocation of molecular patterns associated with pathogens of bacterial origin (bacterial metabolites, bacterial vesicles, and even bacteria) into neurons of the central nervous system, which affects neuronal function through mitochondrial autophagy and ultimately contributes to the development of Parkinson’s disease (Magalhães et al., 2023). Gut microbiota metabolism may lead to altered mitochondrial function or disturbances in mitochondrial quality control, which may alleviate or accelerate the progression of diabetic nephropathy (Ma et al., 2023).

Mitochondrial ROS production, kinetic disorganization, and respiration play important roles in the composition of the gut microbiota (Saint-Georges-Chaumet and Edeas, 2016). Cao et al. used *Lactobacillus plantarum* ZJUIDS14 to intervene in a Non-alcoholic Fatty Liver Disease (NAFLD) model mouse and found that the increase in the relative abundance of Roseburia in the feces of the model mice also increased Drp 1 and decreased the mitochondrial oxidative phosphorylation (OXPHOS) thereby improving mitochondrial function and ultimately attenuating high-fat diet-associated hepatic steatosis, and liver injury (Cao et al., 2022). Drp1 induces ROS accumulation in IECs potentially leading to aberrant composition of the anaplastic bacillus population and reduced production of SCFAs (Duan et al., 2020). In contrast, inhibition of Drp1 by P110 treatment reduced TNF-α or H2O2-induced mitochondrial damage and prevented demyelination in EAE-immunized mice (Luo et al., 2017). It was found that mtDNA defects as well as changes in mitochondrial structure and function can modulate gut microbiota to influence MS progression (Mahad et al., 2008). For example, other mouse strains carrying different mtDNA variants such as C57BL/6, ND6 P25L/(C57BL/6 J), and mt-129(C57BL/6 J) regulate the composition of the gut microbiota by altering the mitochondrial redox state and the associated ROS production (Yardeni et al., 2019). Similarly, mitochondria can produce ROS during OXPHOS respiration, and studies using mice carrying different mtDNA variants have found differences in the composition of gut microbiota due to reduced OXPHOS respiration and increased glycolysis (Hirose et al., 2017).

**Figure 1:**
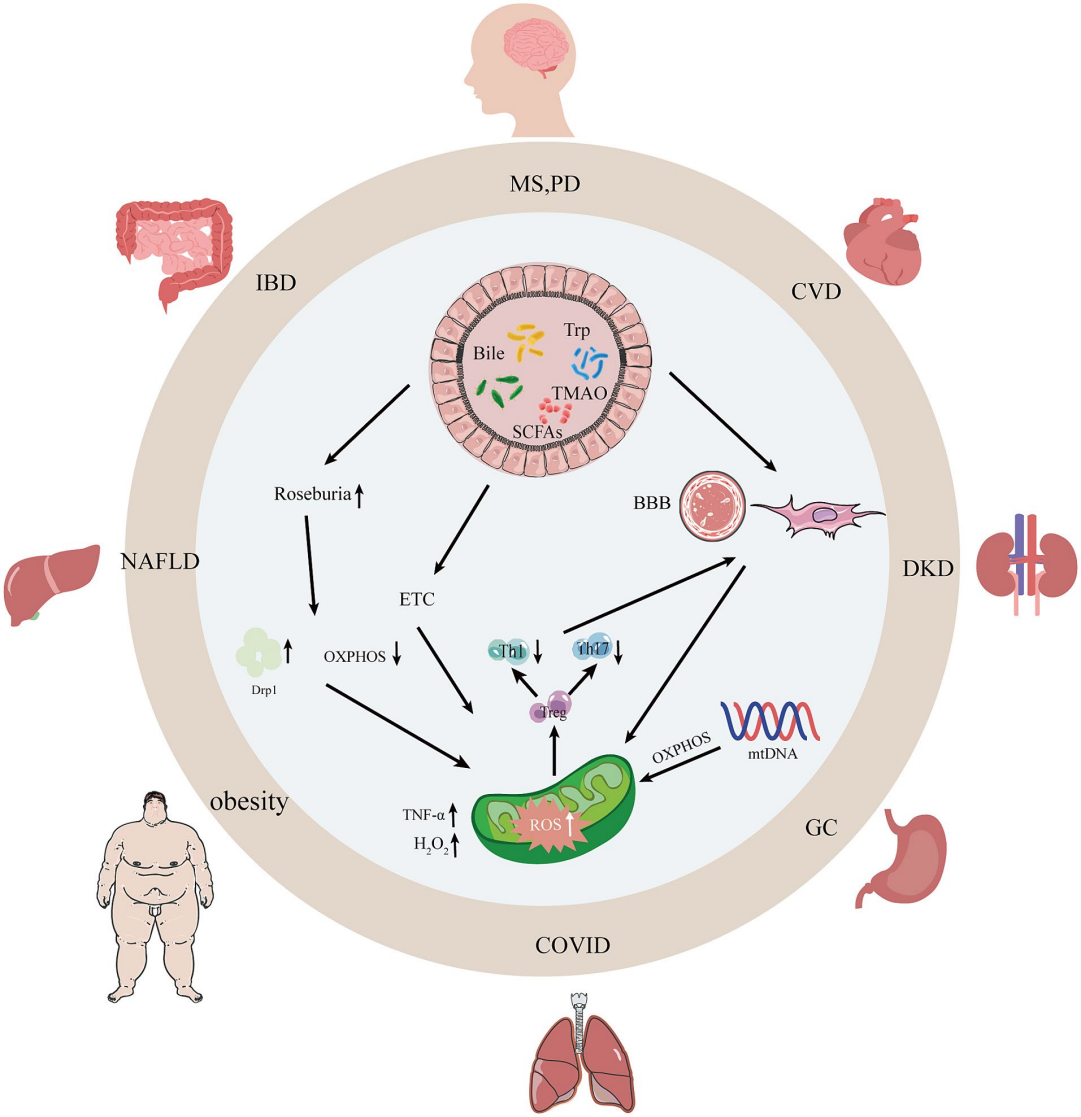
Interactions between gut microbiota, their metabolites, and mitochondria. With the dysregulation of gut microbiota and mitochondrial dysfunction, intestinal occurrences can undergo changes such as leaky gut syndrome, increased intestinal permeability, and intestinal barrier dysfunction thereby causing human diseases. SCFAs, short-chain fatty acids; Bile, bile acids; Trp, tryptophan; TMAO, trimethylamine N-oxide; AMPK, adenylate-activated protein kinase; OXPHOS, oxidative phosphorylation; Drp1, dynamin-associated protein 1; MS, multiple sclerosis; PD, Parkinson’s disease; CVD, atherosclerosis; NAFLD, non-alcoholic fatty liver disease; DKD, diabetic kidney disease; IBD, inflammatory bowel disease.

Thus, these results suggest that establishing the crosstalk between gut microbiota and mitochondria is critical to understanding the complex and evolving interactions between gut microbiota and mitochondria and the host. By targeting the crosstalk between gut microbiota and mitochondria, a range of diseases associated with disorders of gut microbiota and mitochondria can be treated more precisely (e.g., Figure 1).

### 2.4 Gut microbiota and mitochondrial crosstalk in MS

**Figure 2:**
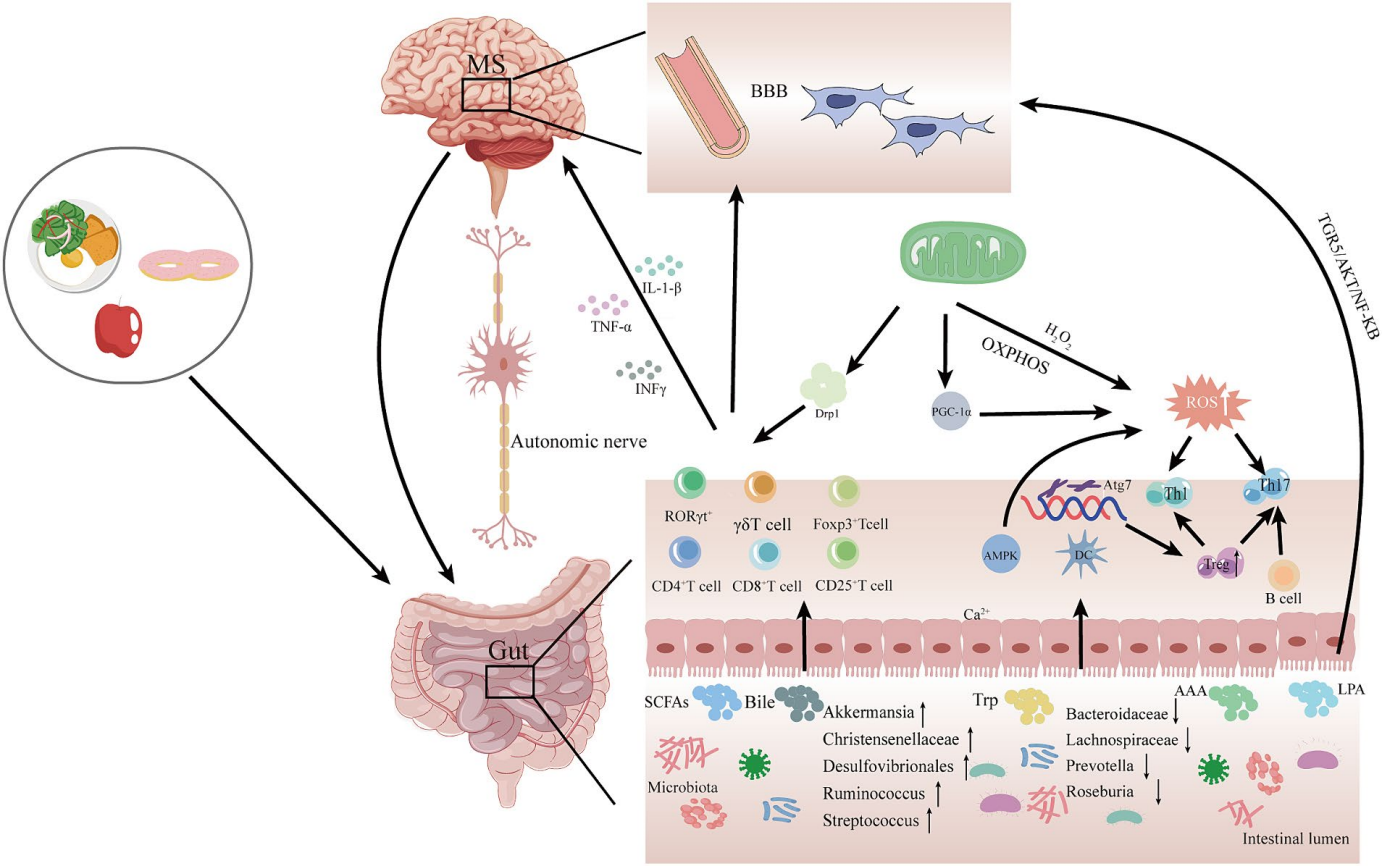
Potential mechanisms for gut microbiota-mitochondrial crosstalk in MS pathogenesis. In this hypothetical crosstalk, the diversity and abundance of gut microbiota change as food is digested, degraded, and absorbed. Subsequently, various gut metabolites, such as SCFAS, bile acids, and Trp, are produced by the gut microbiota, which are transported to the brain via the MGB and interact with mitochondria in the brain. The metabolites of gut microbiota may cause mitochondrial dysfunction, which may attenuate or accelerate the progression of MS. MGB, gut-brain-axis; SCFAs, short-chain fatty acids; Bile, bile acids; Trp, tryptophan; AAA, aromatic amino acids; LSP, lipopolysaccharides; AMPK, adenylate-activated protein kinase; OXPHOS, oxidative phosphorylation; Drp1, dynamin-associated protein 1; DC, dendritic cells; ROS, reactive oxygen species; PGC-1α, peroxisome proliferator-activated receptor γ coactivator; BBB, blood–brain barrier.

As mentioned previously, although both mitochondrial dysfunction and alterations in gut microbiota have been associated with the pathogenesis of MS, it is uncertain whether the gut microbiota transmits signals to the mitochondria to contribute to the development of MS. Therefore, based on the current findings, we propose a potential mechanism by which gut microbiota-mitochondrial crosstalk plays an important role in the pathogenesis of MS (Figure 2). In this crosstalk with the degradation, digestion, and absorption of food by the gut microbiota, the use of probiotics, and fecal transplants, a variety of gut microbiota metabolites are produced in response to the gut microbiota and then enter the circulatory system. These gut microbiota metabolites, such as SCFAs cross the blood–brain barrier to reach the brain and interact with the brain’s mitochondria. Metabolites of gut microbiota may lead to altered mitochondrial function, which may alleviate or accelerate the progression of MS.

## 3 Treatment strategies and future prospects

The etiology of MS involves multiple factors and pathways. In this review, we emphasize the potential mechanisms of gut microbiota and mitochondrial crosstalk in MS progression. To date, several therapeutic strategies, including probiotics, dietary interventions, fecal transplants, and administration of medications, have been used to slow MS progression (Tsogka et al., 2023).

Probiotic and prebiotic supplementation is beneficial in reducing inflammation and modulating immune cytokines (Bronzini et al., 2023). Rahimlou et al. conducted a six-month clinical study on MS patients using probiotic supplements. The results showed a significant increase in brain-derived neurotrophic factor levels and a significant decrease in inflammatory factor IL-6 levels in the probiotic group compared to the placebo group, as well as significant improvements in the General Health Questionnaire (GHQ)-28, Beck Depression Inventory-II, Fatigue Severity Scale and Pain Rating Index (Rahimlou et al., 2022). An intervention study of the probiotic *Saccharomyces boulardii* in 40 MS patients found significant reductions in levels of the inflammatory marker high-sensitivity C-reactive protein along with pain intensity and fatigue, as well as significant improvements in terms of the Quality of Life Scale, and the GHQ Somatic and Social Dysfunction subscales in the probiotic group compared to the placebo group (Asghari et al., 2023). *Palmaria palmata* Alga probiotic intervention in mice in a CPZ model increased Firmicutes/Bacteroidetes ratio, increased the abundance of beneficial Lactobacillus, Proteobacteria, and Bactriodia communities, and prevented CPZ-induced grip strength loss and open field test anxiety, as compared to the control group (Yousof et al., 2023). Anti-inflammatory-antioxidant-rich diet and co-supplemented synbiotics intervention in 69 patients with MS significantly reduced Faecal calprotectin levels at 6 months, reduced intestinal inflammation, and improved clinical manifestations in progressive forms of MS compared with the control group (Moravejolahkami et al., 2023).

The beneficial effects of fasting and ketogenic diets have been demonstrated in several studies in mouse models of EAE by altering the gut microbiota of MS patients through dietary habits (Bai et al., 2021; Brenton et al., 2022; Lin et al., 2023), These dietary interventions delayed the onset of the disease, reducing or even reversing its motor and cognitive symptoms (Guo et al., 2023). Lin et al. found that IF may exert neuroprotective effects on MS patients through several mechanisms, including (1) regulation of blood glucose levels and enhancement of insulin sensitivity; (2) inhibition of inflammatory responses; (3) autophagy activation; and (4) reduction of oxidative stress (Lin et al., 2023). A clinical trial of an anti-inflammatory dietary intervention was conducted in 100 MS patients, and the results of the study showed significant improvements in the Modified Fatigue Impact Scale and the Multiple Sclerosis Quality of Life Scale (mSQoL-54) in the intervention group compared to the control group, while elevated IL-4 levels were observed (Mousavi-Shirazi-Fard et al., 2021).

Fecal microbiota transplantation (FMT) is the most effective gut microbiota intervention currently available (Vendrik et al., 2020). Introducing FMT from MS patients into healthy mice results in reduced Sutterella abundance, decreased anti-inflammatory signaling, and increased EAE inflammation (Berer et al., 2017). Animal studies of mice transplanted with FMT (from normal healthy donors) resulted in slowing the development of EAE, relieving symptoms, improving BBB integrity, and restoring microbiota diversity. Transplantation of FMT from a healthy donor into MS patients improved intestinal motility and motility in patients for at least 2 to 15 years (Zhanel et al., 2023).

Development of drugs targeting mitochondrial dysfunction, e.g., antioxidants targeting mitochondria, including SKQ1 and MitoQ have been tested in preclinical studies (Fields et al., 2023). For example, *in vitro* experiments have shown that SkQ1 was found to accumulate in the mitochondria of oligodendrocytes and microglia and was also found to prevent lipopolysaccharide-induced myelin production in oligodendrocytes could improve MS (Fetisova et al., 2019). Pretreatment and treatment of EAE mice with MitoQ reduces axonal loss and decreases neurological deficits associated with EAE, suggesting that MitoQ has a realized neuroprotective effect (Mao et al., 2013). Mitochondrial transplantation in animal models of MS has recently emerged as a potential therapeutic approach to restore mitochondrial function in diseased cells (Clemente-Suárez et al., 2023). However, methods involving the use of mitochondrial transplants to slow or block axonal degeneration require additional *in vitro* or *in vivo* studies for validation (Picone and Nuzzo, 2022).

As mentioned previously, mtDNA variants would be potential co-regulators of mitochondrial function and gut microbiota. Therefore, regulating pathology-associated mtDNA variants may be a potential approach to control MS. Currently, there are several methods to manipulate mtDNA, including transcription activator-like effector nucleases (TALENs) and zinc finger nucleases (mtZFN; Gammage et al., 2018). mitoTALENs have been designed to specifically cleave certain sequences in mtDNA in order to remove mtDNA containing deleterious point mutations (Hashimoto et al., 2015). Correcting mtDNA heterogeneity through the use of mtZFN may be useful as a treatment for a variety of genetically caused heterogeneous mitochondrial diseases (Gammage et al., 2018).

Probiotic use, dietary interventions, fecal transplants, mtDNA variant modulation, drug administration, and mitochondrial transplants would be potential co-regulators of mitochondrial function and gut microbiota. Thus, modulation of these factors associated with pathology may be a potential approach to treating MS based on gut microbiota-mitochondrial crosstalk. In summary, intestinal flora and mitochondrial crosstalk are important in the development of MS, and therefore, we propose a potential therapeutic approach based on the presence of this crosstalk in MS. Unfortunately, however, no specific targeted drugs that can modulate this crosstalk have been identified in clinical studies. It is hypothesized that supplementation with probiotics, prebiotics, dietary fibers (SCFAs), and an anti-inflammatory diet can be beneficial in the treatment of MS and correlate with gut flora-mitochondrial crosstalk. And in order to assess the importance of gut flora-mitochondrial crosstalk as a biomarker of MS and the efficacy of flora interventions, translational approaches and relevant clinical trials are necessary. These are needed to better understand the temporal and causal relationships between gut flora-mitochondrial crosstalk and the development of MS.

## 4 Conclusion

This paper reviews the close relationship between gut flora and mitochondria during MS by modulating gut flora composition, metabolites produced by gut flora and mitochondrial dysfunction to reduce ROS-related inflammation and attenuate neural demyelination changes in MS patients. Although much progress has been made so far in exploring the effects of gut flora and mitochondrial function on MS, we still do not have enough information to fully elucidate the impact of the gut flora-mitochondrial crosstalk on the regulatory function of MS. In the future, we will conduct more studies to elucidate the mechanism of gut flora-mitochondrial crosstalk in MS, which will provide important evidence to ameliorate or delay the symptoms of MS.

## Author contributions

HT: Conceptualization, Methodology, Writing – original draft. DH: Conceptualization, Methodology, Writing – original draft. JW: Investigation, Validation, Writing – review & editing. HL: Investigation, Validation, Writing – review & editing. JG: Investigation, Validation, Writing – review & editing. YZ: Investigation, Validation, Writing – review & editing. LX: Investigation, Validation, Writing – review & editing. AZ: Supervision, Writing – review & editing. XK: Supervision, Writing – review & editing. ZL: Supervision, Writing – review & editing.

## Glossary

MS: multiple sclerosis
CNS: central nervous system
DMT: disease-modifying therapies
MGB: gut-brain-axis
ATP: Adenosine triphosphate
ROS: reactive oxygen species
ETC: electron transport chain
PGC-1alpha: peroxisome proliferator-activated receptor γ coactivator 1-alpha alpha
EAE: autoimmune encephalomyelitis
MARK: mitogen-activated kinase
IF: intermittent fasting
CPZ: cuprizone
Tregs: regulatory T cells
SCFAs: short-chain fatty acids
Trp: tryptophan
BBB: blood–brain barrier
TUDCA: taurine deoxycholic acid
GPBAR1: G protein-coupled bile acid receptor 1
TGR5/AKT/NF-KB: G-protein coupled bile acid receptor Gpbar1/ threonine kinase/ nuclear factor κB
AAA: aromatic amino acids
Drp1: dynein-associated protein 1
TNFα: tumor necrosis factor-α
OXPHOS: mitochondrial oxidative phosphorylation
