# Supplementary material for: The role of the “gut microbiota-mitochondria” crosstalk in the pathogenesis of multiple sclerosis
Source: Front Microbiol. 2024 Apr 29;15:1404995. doi: 10.3389/fmicb.2024.1404995 (PMC11089144; doi:10.3389/fmicb.2024.1404995)
Supplement: Supplementary file 1 [file Table_1.docx]

Table 1 Characteristics of intestinal flora in MS patients.

| Study ID | Participants | Intestinal flora diversity | Upregulated microbiota | Downregulated microbiota | Sequencing method |
| --- | --- | --- | --- | --- | --- |
| Cantare et al.,2015 | Patient:15  Healthy Patient:8 | - | *Bacteroidaceae*, *Faecalibacterium* | *Ruminococcus* | 16S rRNA |
| Miyake et al.,2015 | Patient:20  Healthy Patient:40 | α diversity index was not statistically significant | *Anaerostipes sp.*, *Faecalibacterium*, *Bacteroides*, *Prevotella* | *Bifidobacterium*, *Streptococcus* | 16S rRNA (V1-V2 region) |
| Chen et al.,2016 | Patient:31  Healthy Patient:36 | α diversity index was not statistically significant | *Adlercreutzia*, *Prevotella*, *Parabacteroides* | *Psuedomonas*, *Mycoplana*, *Haemophilus*, *Blautia*, *Dorea* | 16S rRNA (V3-V5 region) |
| Jangi et al.,2016 | Patient:60  Healthy Patient:43 | α diversity index was not statistically significant | *Butyricimonas* | *Akkermansia*, *Methanobrevibacter* | 16S rRNA (V3-V5 region) |
| Tremle et al.,2016 | Patient:18  Healthy Patient:17 | α diversity index was not statistically significant | *Lachnospiraceae*, *Ruminococcaceae* | *Bifidobacterium*, *Desulfovibrionales*, *Christensenellaceae* | 16S rRNA (V4 region) |
| Cekanaviciute et al.,2017 | Patient:71  Healthy Patient:71 | α diversity index was not statistically significant | *Parabacteroides* | *Acinetobacter*, *Akkermansia* | 16S rRNA (V3-V5 region) |
| Cosorich et al.,2017 | Patient:19  Healthy Patient:17 | α diversity index was not statistically significant | *Prevotella* | - | - |
| Forbes et al.,2018 | Patient:19  Healthy Patient:23 | α diversity index was statistically significant | *Lachnospiraceae*, *Gemmiger sp*, *Sporobacter* | *Actinomyces*, *Clostridium III*, *Eggerthella*, *Faecalicoccus*, *Streptococcus* | 16S rRNA (V4 region) |
| Kozhieva et al.,2019 | Patient:15  Healthy Patient:15 | α diversity index was statistically significant | - | *Actinomycetales*, *Desulfovibrionales*, *Ruminococcaceae*, *Verrucomicrobiales*, *Gemmiger sp* | 16S rRNA (V3-V4 region) |
| Oezguen et al.,2019 | Patient:13  Healthy Patient:14 | α diversity index was not statistically significant | *Succinivibrio* | *Butyricicoccus*, *Clostridium III*, *Coprococcus*, *Escherichia*/*Shigella*, *Ruminococcus*, *Dorea*, *Parabacteroides*, *unclassified Coriobacteriaceae* | 16S rRNA(V3-V5 region) |
| Storm-Larsen, et al.,2019 | Patient:36  Healthy Patient:165 | α diversity index was not statistically significant | *Faecalibacterium* | - | 16S rRNA (V3-V4 region) |
| Ventur et al.,2019 | Patient:45  Healthy Patient:44 | α diversity index was not statistically significant | - | *Akkermansia* | 16S rRNA (V4 region) |
| Zeng, et al.,2019 | Patient:34  Healthy Patient:34 | α diversity index was not statistically significant | *Prevotella* | *Streptococcus* | 16S rRNA (V3-V4 region) |
| Ling et al.,2020 | Patient:22  Healthy Patient:33 | α diversity index was not statistically significant | *Bilophila*, *Butyricicoccus*, *Clostridium III*, *Faecalibacterium*, *Haemophilus*, *Dorea*, *Roseburia*, *Gemella*, *Granulicatella* | *Flavonifractor*, *Blautia* | 16S rRNA (V3-V4 region) |
| Choileái et al.,2020 | Patient:26  Healthy Patient:39 | The Shannon index was significantly reduced in MS patients | *Clostridium III*, *Coprococcus*, *Paraprevotella*, *Methanobrevibacter* | *Ruminococcaceae* | 16S rRNA (V4 region) |
| Reynders et al., 2020 | Patient:98  Healthy Patient:120 | α diversity index decreased | *Butyricicoccus* | *Akkermansia*, *Clostridium III*, *Parabacteroides*, *Gemmiger sp*, *Sporobacter* | 16S rRNA (V4 region) |
| Saresella et al.,2020 | Patient:38  Healthy Patient:38 | α diversity index was not statistically significant | *Coprococcus*, *Blautia*, *Parabacteroides*, *Roseburia* | *Collinsella*, *Eubacterium* | 16S rRNA (V3-V4 region) |
| Castillo-Álvarez, et al.,2021 | Patient:15  Healthy Patient:14 | α diversity index was not statistically significant | *Eubacterium*, *Prevotella*, *Uncultured Bacteroides*, *Uncultured alpha Proteobacterium*, *Uncultured Pseudomonas sp* | *Alistipes onderdonkii*, *Anaerostipes sp*. *Clostridium III*, *Coriobacterium sp.*, *Faecalibacterium*, *Ruminococcus*, *Bifidobacterium*, *Uncultured Oscillospiraceae bacterium*, *Uncultured Blautia sp*, *Uncultured Sinorhizobium sp.*, *Uncultured Dialister sp* | 16S rRNA (V4 region) |
| Galluzzo, et al.,2020 | Patient:15  Healthy Patient:15 | α diversity index was not statistically significant | *Bacteroidaceae*, *Lachnospiraceae*, *Tannerellaceae*, *Rikenellaceae* | *Akkermansiaceae*, *Clostridiales*, *Desulfovibrionaceae*, *Family XIII*, *Christensenellaceae*, *Ruminococcaceae* | 16S rRNA (V3-V4 region) |
| Takewak et al.,2021 | Patient:118  Healthy Patient:55 | α diversity index was not statistically significant | *Alistipes onderdonkii*, *Megamonas*, *Roseburia* | *Bifidobacterium*, *Streptococcus* | 16S rRNA (V1-V2 region) |
| Cox et al.,2021 | Patient:243  Healthy Patient:40 | α diversity index was not statistically significant | *Anaerococcus*, *Blautia*, *Dorea* | *Akkermansia*, *Clostridaceae*, *Clostridium III*, *Enterobacteriaceae*, *Ruminococcaceae* | 16S rRNA (V4 region) |
| Cantoni et al.,2022 | Patient:24  Healthy Patient:25 | α diversity index was not statistically significant | *Anaerostipes sp.*, *Faecalibacterium*, *Prevotella*, *Lachnospiraceae* | - | 16S rRNA (V1-V3 region), Metagenomic whole-genome shotgun approach |
| Mekky et al.,2022 | Patient:30  Healthy Patient:20 | - | *Prevotella* | **B. fragilis**, *C. perfringes* | 16S rRNA |
| Vacaras, et al.,2023 | Patient:50  Healthy Patient:21 | α diversity index was not statistically significant | *Faecalibacterium*, *Prevotella*, *Bifidobacterium* | - | 16S rRNA (V3-V4 region) |

Table 2 A summary of current studies about the impact of gut microbial metabolites in MS in vivo and in vitro.

| Metabolites | Human/animal/cell type species | Conclusions | Reference and year |
| --- | --- | --- | --- |
| bile | MS patients | a decrease in the abundance of microbes involved in fatty acid metabolism (bile metabolism) | Chen et al.,2016 |
| Trp | MS patients | The QA/KA ratio was higher in MS groups compared to controls | Lim et al.,2017 |
| Trp | MS patients | each 1 mcg/mL increase in serum Trp level was associated with a 20% and 32% decrease in adjusted odds of having MS. | Nourbakhsh et al.,2018 |
| SCFAs（acetate, propionate, and butyrate） | MS patients | A depletion of acetate, propionate, and butyrate was observed in MS. SCFA level was positively correlated with pTreg frequency. | Zeng et al.,2019 |
| SCFAs(butyrate) | C57BL/6 J (B6) mice | butyrate treatment suppressed demyelination and enhanced remyelination | Chenet al.,2019 |
| butyricum | C57BL/6J WT mice | C. butyricum treatment reduced Th17 response and increased Treg response in the gastrointestinal tract and extra-gastrointestinal organ systems in EAE mice. | Chen et al.,2019 |
| SCFAs（acetate, butyrate, and valerate） | MS patients | In MS patients, butyrate was higher and acetate lower. Acetate levels are associated negatively with IFNG. IFNG and TNF were favorably linked with butyrate and valerate. | Olsson et al.,2019 |
| SCFAs(acetate, propionate, and butyrate) | MS patients, IL-10−/− mice | SCFAs (acetate, propionate, and butyrate) are significantly decreased in MS.  SCFAs increase IL-10 T cells，Th17 and Th1 effector cells | Park et al.,2019 |
| SCFAs(propionate) | MS patients | MS exhibited reduced PA amounts. T cells increased, whereas Th1 and Th17 cells decreased after PA intake. | Duscha A et al.,2020 |
| SCFAs (acetate) | MS patients | acetate levels were higher in patients, and an inverse correlation exists between acetate levels and naïve CD4+ T lymphocytes, while a direct correlation exists with IL-17-producing CD8+ T cells. | Pérez-Pérez et al.,2020 |
| SCFA (butyric acid (BA), Caproic acid (CA)) | MS patients | In MS, the concentration of BA was reduced and that of CA was increased. the higher plasma concentrations of lipopolysaccharide and intestinal fatty acid-binding protein. CA was positively associated with CD4+/IFNγ+ T lymphocytes, and the BA/CA ratio correlated positively with CD4+/CD25high/Foxp3+ and negatively with CD4+/IFNγ+ T lymphocytes. | Saresella et al.,2020 |
| SCFA （butyrate） | MS patients | The microorganisms that produce butyric acid are reduced in MS. The abundance of butyrate correlated positively with the levels of chemokines such as IL-8 and MIP-1a, while it correlated negatively with those of inflammatory cytokines such as TNF-α. | Ling et al.,2020 |
| Trp | MS patients | MS patients had a significantly lower urine concentration of kynurenine than healthy controls. | Gaetani et al.,2020 |
| Bile acids（TUDCA） | MS patients | Multiple bile acid metabolites were found in smaller amounts in MS, and TUDCA supplementation ameliorated neuroinflammation in EAE through its effects on GPBAR1. | Bhargava et al.,2020 |
| SCFAs (propionate acid) | MS patients | An increase of functionally competent regulatory T (Treg) cells in MS, whereas Th1 and Th17 cells decreased significantly after PA intake. | Duscha et al.,2020 |
| SCFAs (butyrate and propionate) | MS patients | A reduction in butyrate and propionate biosynthesis and corresponding metabolic changes in MS. | Takewak et al.,2020 |
| AAA metabolites | MS patients | AAA metabolism results in the reduced production of immunomodulatory metabolites and increased production of metabotoxins in MS. | Fitzgerald et al.,2021 |
| SCFAs (butyrate, valerate ) | MS patients | MS patients had increased butyrate levels, and butyrate and valerate correlated positively with proinflammatory cytokines (IFNG and TNF). SCFAs were inversely connected with clinical impairment. | Olsson et al.,2021 |
| SCFAs (butyrate) | MS patients | MS has reduced indolelactate and its producing bacteria and lower levels of species and genes involved in butyrate production. | Levi et al.,2021 |
| SCFAs (butyrate) | C57BL/6JOlaHsd mice | the butyrate was increased and ameliorated the Clinical Signs and Reduced CNS Autoimmunity in EAE Mice | Calvo-Barreiro et al.,2021 |
| SCFAs (propionate) | MS patients | the propionate was reduced in MS patients | Trend et al.,2021 |
| LPS | MS patients | MS participants had higher breakdown of LPS molecules, but lower resistant starch metabolism. | Mirza et al.,2022 |
| SCFAs | MS patients | MS patients had decreased SCFAs levels | Cantoni et al.,2022 |
| tryptophan metabolism | PWD/PhJ (PWD) mice | The number of IL-17 and IFNγ-producing CD4+ T cells was also reduced with low tryptophan, L. reuteri colonization elevated the number and frequency of TCRγδ cells and their production of IL-17, in the presence of high dietary tryptophan. | Montgomery et al.,2022 |
| Bile acids （TUDCA） | C57BL/6 mice | TUDCA suppressed excessive activation of astrocytes and inhibited inflammatory responses in the cerebral cortex of EAE mice via TGR5/AKT/NFκB signaling pathway. | Xu et al.,2023 |
| Bile acids （TUDCA） | MS patients | Central memory CD4+ and Th1/17 cells decreased, while CD4+ naïve cells increased in the TUDCA arm compared to placebo. | Ladakis et al.,2024 |

Trp: altered tryptophan; SCFAs: short-chain fatty acids; AAA: aromatic amino acid; LSP: Lipopolysaccharide; TUDCA: effects of a secondary bile acid; GPBAR1: G protein-coupled bile acid receptor
